# The impact of social distancing on COVID-19 infections and deaths

**DOI:** 10.1186/s40794-021-00137-3

**Published:** 2021-05-04

**Authors:** André de Souza Melo, Ana Iza Gomes da Penha Sobral, Marcelo Luiz Monteiro Marinho, Gisleia Benini Duarte, Amanda Aires Vieira, Marcos Felipe Falcão Sobral

**Affiliations:** 1 Departamento de Economia, Federal Rural University of Pernambuco, Avenida Dom Manoel de Medeiros, s/n – Dois Irmãos, Recife, PE Brazil; 2 Departamento de Psicologia Cognitiva, Federal University of Pernambuco, Av. Prof. Moraes Rego, 1235 - Cidade Universitária, Recife, PE 50670-901 Brazil; 3 Faculdade Boa Viagem, R. Jean Emile Favre, 422 - Ipsep, Recife, PE 51190-450 Brazil

**Keywords:** COVID-19, Social isolation, Quarantine, SARS-CoV-2

## Abstract

**Background:**

To assess the impact of the social isolation index on the number of infections and deaths by COVID-19 in the state of São Paulo (Brazil).

**Methods:**

Daily isolation data, obtained through geolocation information by mobile phone, were evaluated together with the number of daily infections and deaths by COVID-19 in the state of São Paulo. The study was conducted from February 26 to May 19, 2020. The data were modeled through the vector autoregression (VAR) model.

**Results:**

The isolation index has an effect of approximately 5% in variation in the number of infections, and 7% in the number of deaths. The impulse response function (IRF) caused a drop of 0.15% in the number of new cases/day, and 0.17% in the number of deaths/day following a shock in the isolation index. For both cases, this effect occurred 1 day after the shock and stabilized after 10 periods. An increase of 1% in the isolation index led to a reduction of 6.91% in new cases and 6.90% in the number of deaths. The 30 cumulative day reduction reached 22.72% in terms of transmission and 35.39% for deaths.

**Conclusions:**

The social isolation index is related to deaths and infections from SARS-CoV-2. Although distancing measures are accompanied with impacts on the economy and the emergence of other morbidities, the benefits caused by the reduction in the speed of contagion are significant. The adoption of distancing measures has a substantial impact on the number of infected individuals and deaths by COVID-19.

## Background

The outbreak of COVID-19 began in December 2019 in Wuhan, China, and has spread rapidly worldwide. In March 2020, the World Health Organization (WHO) declared the outbreak a pandemic amid the increasing numbers of cases and deaths. By mid-May, more than 4,993,470 people were infected, and more than 327,738 had died of the virus [32].

In Brazil, the epidemic was declared a public health emergency on February 3 [25]. Faced with a growing number of infected people, authorities in the country adopted measures to lessen social contact to slow the spread of the virus, such as by temporarily closing schools, shops, restaurants, and bars; prohibiting public events; and promoting or imposing work at home. Law 13.979 gave authorities the power to implement public quarantine measures as well as other actions deemed necessary to control the spread of the virus [5, 8].

São Paulo has been among the country’s states with the greatest number of COVID-19 cases and deaths. According to data from the government of São Paulo, as of May 30, 2020, there were 101,556 confirmed cases and 7275 deaths due to COVID-19 in the state [28]. In May 2020, São Paulo completed 2 months of quarantine to contain the spread of the virus throughout the state. The 60-day cycle was completed during a period of higher disease progression in cities in the interior, with almost 90% occupation of hospital beds in Greater São Paulo.

Some studies have reported a relationship between containment measures and the number of people infected by COVID-19. Kucharski [20] and Wang [13] showed that travel restrictions may reduce the spread of the contagion. Berger [3] emphasized the impact of increased testing and directed quarantine in connection with a decline in COVID-19 cases. In addition, studies have demonstrated the sensitivity of the contagion to social distancing measures [13, 22].

The initiation and duration of various interventions can influence the disease’s impact on the current epidemic pattern [16]. Hence, the early adoption of quarantine measures and social isolation can have a large effect on the spread of the epidemic. Late implementations may not be effective at reducing the number of cases, as wide dissemination of the disease would have already occurred.

Preliminary studies have established a relationship between lockdown measures in China and mortality and infection due to COVID-19: Social containment lowered the incidence of the disease and mortality rates [11]. Similar results were obtained by Friedson [15] in the U.S. state of California, who identified a significant drop in COVID-19 cases 1 month after the state’s shelter-in-place order was implemented.

In China, actions such as quarantine, social distancing, and isolation have shown that they may interfere with the rate at which COVID-19 spreads, thus helping to contain the spread of the disease [2]. However, in Brazil, there is still little evidence on the effect of social distancing on the disease’s spread. Even in other countries, studies using information technology and geolocation as an indicator of social isolation have been scarce. In this context, this study sought to investigate the relationship between the number of cases and deaths caused by SARS-CoV-2 and the social isolation index in the state of São Paulo.

## Methods

### Data collection

The social isolation index data used in this study was provided by the In Loco Company for the period between February 26 to May 19, 2020. This index is indicated using the daily percentage of mobile devices that have remained in people’s homes. The company uses aggregated data, with user consent, and does not collect personally identifiable information from users [19]. The authors obtained consent to use In Loco’s database of social isolation data through data transfer cooperation.

Information on the number of COVID-19 cases and deaths were extracted from the Coronavirus Dashboard of the Ministry of Health [9].

Both geolocation and epidemiological data on the state of São Paulo were collected. This region was chosen as it is the most populous state in the country, with nearly 46 million inhabitants [18]. Furthermore, the first registered case of COVID-19 (and community transmission) in Brazil was recorded in São Paulo.

The isolation data were aggregated without differentiating for gender. Thus, there was no analysis by gender in this study.

### Model

This study aimed to analyze the impact of variation in the social isolation index on the number of cases and deaths due to COVID-19. Data were modeled using the vector autoregression (VAR) model, as described in Eq. 1,

1 $$ {y}_t^{\prime }\ {A}_0=\sum \limits_{l=1}^p{y}_{t-1}^{\prime }\ {A}_l+{\varepsilon}_t^{\prime }\  for\ 1\le t\le T $$

where ${y}_t^{\prime }$ is an *n x* 1 vector of endogenous variables; *A*_0_ is an *n x n* array of parameters; *A*_*l*_ is an *n x n* array of parameters of the lagged variables, for 1 ≤ *l* ≤ *p*; *ε*_*t*_ is an *n x* 1 vector of structural shocks; *p* is the lag order; and *T* is the size of the sample. The structural model presented in Eq. (1) was not determined. Thus, to estimate the VAR, it was necessary to use a reduced form, pre-multiplying *A*^−1^ and obtaining Eq. 2,

2 $$ {y}_t^{\prime }={y}_{t-1}^{\prime }\ B+{u}_t^{\prime } $$

where $B={FA}^{-1}\ {u}_t^{\prime }={\varepsilon}_t^{\prime }\ {A}^{-1}$ and $E\left[{u}_t^{\prime }\ {u}^t\right]=\Omega ={\left({AA}^{\prime}\right)}^{-1}$ is a variance-covariance matrix of the residuals. According to Sims [16], to estimate Eq. (2), one must identify Eq. (1) by restricting the array of contemporaneous effects *A*_0_ through the Cholesky decomposition. Hence, it was possible to recover the structural parameters of the first equation after estimating the second.

To restrict contemporaneous effects, we assumed *A*_0_ as a lower triangular matrix, that is, the number of cases and deaths due to COVID-19 would have contemporaneous effects on the isolation index. However, the isolation index had no contemporaneous effect on the number of cases and deaths. The empirical model has the structural form defined in Eq. 3,

3 $$ {y}_t={\left({Cases}_t, Isolation\ {Index}_t\right)}^{\prime } $$

where *Cases*_*t*_ is the number of new cases every day in the state of São Paulo and *Isolation Index*_*t*_ is the daily rate of isolation for the same region. The model was estimated using Eq. 4.

4 $$ \left[\begin{array}{cc}1& 0\\ {}{a}_{12}& {a}_{22}\end{array}\right]\left[\begin{array}{c}{Cases}_t\\ {}\  Isolation\ {Index}_t\ \end{array}\right]=\left[F\right]\left[\begin{array}{c}{Cases}_{t-1}\\ {}\  Isolation\ {Index}_{t-1}\ \end{array}\right]+{C}_{\xi } $$

Separately, another model was estimated considering the daily number of deaths due to COVID-19 as exogenous variables, generating the following equations,

5 $$ {y}_t={\left({Deaths}_t, Isolation\ {Index}_t\right)}^{\prime } $$

6 $$ \left[\begin{array}{cc}1& 0\\ {}{a}_{12}& {a}_{22}\end{array}\right]\left[\begin{array}{c}{Deaths}_t\\ {}\  Isolation\ {Index}_t\ \end{array}\right]=\left[F\right]\left[\begin{array}{c}{Deaths}_{t-1}\\ {}\  Isolation\ {Index}_{t-1}\ \end{array}\right]+{C}_{\xi } $$

where *Deaths*_*t*_ is the daily number of deaths in the state of São Paulo.

Three endogenous variables were defined for Eqs. 4 and 6, the first being the constant. The second indicates the day on Sunday, the day in which the isolation index has typically had higher values. Finally, the third variable, the temporal dummy, indicates the quarantine policy in the state of São Paulo.

After estimating the VAR, the reduced form of Eq. 2 was placed as dependent on the residuals. The estimated parameters were then used to identify how the variables responded to shocks in *u*_*t*_. The results of this procedure is called variance decomposition and impulse response function (IRF).

According to Enders [12], variance decomposition signals how much information (the variance of the forecast error) an endogenous variable contributes to the other variables in a model. The IRF determines how an exogenous variable can be explained when exogenous shocks occur in other variables.

In the models we estimated, the variance decomposition showed how the isolation index influenced the variation in the number of contaminants and deaths due to COVID-19. The IRF demonstrated how the number of cases and deaths responded to an increase in the isolation index.

To test for the presence of non-stationarity, a condition for the VAR, three unit root tests were performed: the Augmented Dickey Fuller test, the Kwiatkowski–Phillips–Schmidt–Shin test, and the Phillips–Perron test.

## Results

The temporal series of infections and deaths by COVID-19 and social isolation were stationary, as verified using the Phillips–Perron test. These variables had the same structural breaks in March 2020, where the number of cases and deaths showed an upward trajectory. During March, distancing and isolation measures were also adopted [28].

The social isolation index for São Paulo was, on average, about 33% of individual devices remained at home before the state’s quarantine policy. This proportion did not change even when the first case of COVID-19 was reported on February 26. Only on March 22, following the lifting of quarantine restrictions, did the social isolation index change to 51% of devices at home and the average rose to 48%.

Akaike’s information criterion was used to determine the order of the VAR lag. The results suggest that the estimated model presented two lags. Table 1 depicts the decomposition of variance for the number of new cases in São Paulo in Model 1 and the number of deaths in Model 2. The variance decomposition explains how social isolation can influence the number of case and death variations over time.

**Table 1:** Variance decomposition for the number of cases (Model 1) and deaths (Model 2)

Time	Model 1		Model 2	
	Cases	Isolation Index	Deaths	Isolation Index
**1**	100.000	0.000	100.000	0.000
**10**	94.945	5.055	92.828	7.172
**20**	95.006	4.994	92.793	7.207
**30**	95.007	4.993	92.793	7.207

For Model 1 (COVID-19 cases), in the first moment, any variation in the number of cases was due to the characteristics of the series, and the isolation index had no effect on new cases. Over time, the isolation index had an effect of approximately 5% in the variation of the number of infections. For Model 2 (COVID-19 deaths), the isolation index accounted for 7% of the variation in the daily number of deaths.

**Fig. 1:**
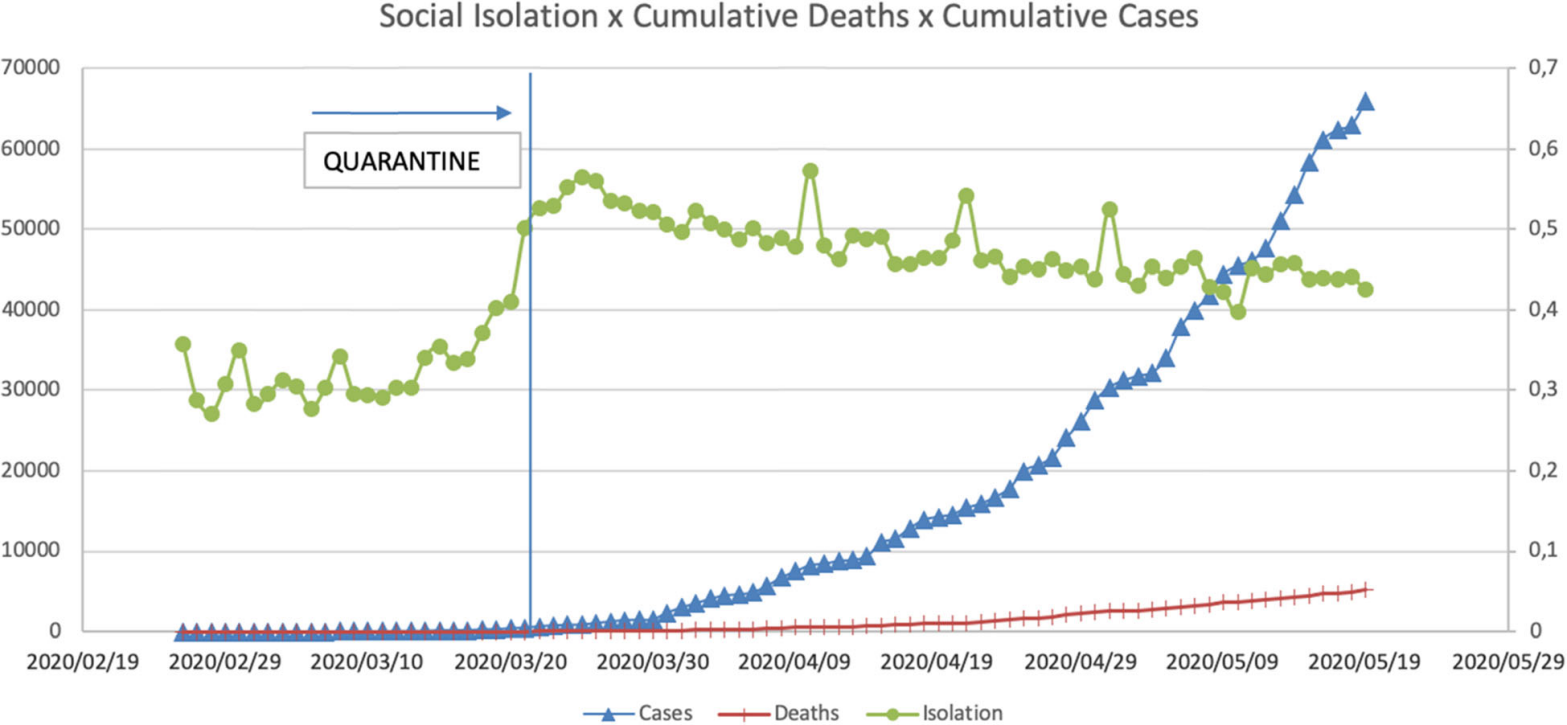
Response regarding the number of cases to the isolation shock index

Figure 1 shows the impulse response function (IRF) that describes how the number of COVID-19 cases responded to an increase in social isolation. The response to a shock in the isolation index caused a 0.23% drop in the number of new cases per day. This response was observed 1 day after the shock. After this effect occurred, the number of new cases tended to increase, returning to stability after the tenth day. The outcome reveals a short-term effect of variation after the shock. The blue line indicates the confidence interval calculated using a Monte Carlo simulation.

**Fig. 2:**
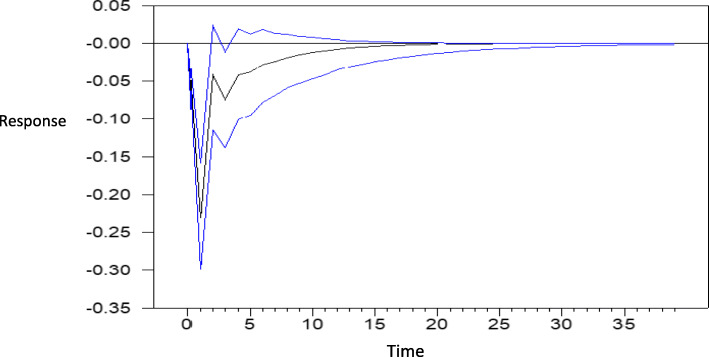
Response of number of deaths to isolation shock index

Figure 2 shows how the number of deaths due to COVID-19 responded to an increase in the social isolation index. The IRF demonstrated that an increase in the isolation index resulted in a reduction of 0.17% in the number of deaths. As in the previous model, this effect occurred 1 day after the shock and stabilized after 10 periods.

**Fig. 3:**
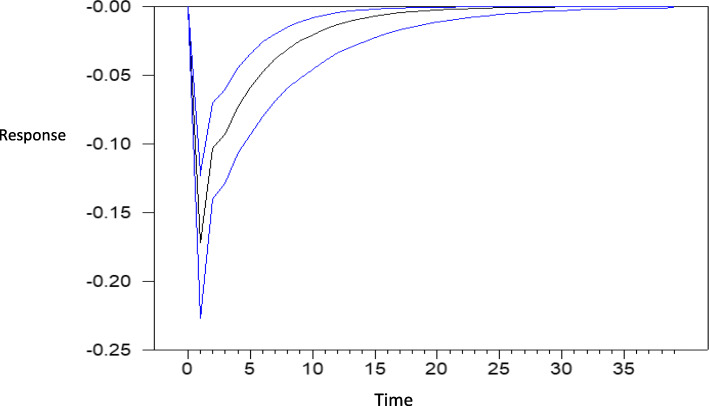
Cumulative cases, cumulative deaths, and isolation index for São Paulo

To better explain the IRF, Table 2 portrays the effect of a 1% increase in the isolation index for the number of cases and deaths due to COVID-19. The values presented in the table were extracted and normalized from the IRF in Figs. 1 and 3. For the transmission of COVID-19, the increase of 1% led to a 6.9% reduction in the number of cases. The cumulative effect embodies the evolution of the response and a continuing decline in the number of cases. The number of deaths had a similar outcome, with the cumulative effect being larger with respect to the number of cases.

**Table 2:** Responses regarding the number of cases and deaths to a 1% isolation shock (%)

Variable	Maximum Response	Accumulated Response (10 days)	Accumulated Response (20 days)	Accumulated Response (30 days)
Cases	−6.91	−16.11	−20.26	−22.72
Deaths	−6.90	−27,03	−33,25	−35,39

## Discussion

As a non-pharmacological strategy to combat COVID-19, governments worldwide have adopted some level of intervention in terms of social habits. These actions have been in the form of quarantines, isolation, or social distancing measures.

Quarantine involves separating suspect persons from those that are not sick, while isolation entails separating those who are already sick or contaminated [33]. Social distancing aims to prevent sick people from coming into close contact with healthy people to reduce opportunities for disease transmission [27]. These may include large-scale actions, such as by canceling group events or closing down public spaces, as well as individual decisions, such as by avoiding crowds [27].

Amid the ongoing pandemic, several countries have implemented strict social restrictions, reducing movement outside people’s homes to an absolute minimum, except for essential workers [17], to contain viral transmission. Thus, we sought to identify the connection between social isolation and the number of cases and deaths caused by COVID-19 in the state of São Paulo, considering that Brazil and the findings point to a significant relationship.

Prior to the occurrence of community transmission in Brazil, in February 2020, changes to legislation were carried out, allowing for the adoption of quarantines, isolation, and even compulsory testing [5].

The state of São Paulo has employed harsh isolation measures, attaining indices that influenced the reduction of cases and deaths recorded daily: 59% in April and 48–50% in May [28]. However, to ensure that such steps are successful, a few factors must be considered, especially the public’s fulfillment of imposed measures and awareness of their importance [24].

Briscese et al. [6] reported that presenting the beginning and end dates of these actions also contributes to the success of their execution. In the United States, these factors seem to relate to local income, partisanship, and political beliefs, and in Israel, with a probable loss of revenue [4, 26].

Flaxman et al. [14] showed that in countries that have adopted severe restrictive measures, including lockdowns, there have been a significant drop in the number of infections, with an attenuation of more than 50% regarding the contagion. We confirmed these findings. Social isolation was responsible for 5% of variations in infections and 7% of deaths in the state of São Paulo. In addition, lockdowns have shown promising results. A study in China indicated that more rigorous confinement of persons in high-risk areas has the potential to reduce the spread of COVID-19 [21].

Courtemanche et al. [10] explained the impact of social distancing policies in the United States following more stringent measures of social restriction. They observed a 3% reduction in the growth rate of COVID-19 cases six to 10 days after the onset; this figure remained constant at 8.6% after the 21st day. Although the data obtained were from a different location, our research corroborates this study, as it has identified that the effect of social isolation has a 10-day duration.

Despite the reduction in cases, both lockdowns and social distancing may lead to the appearance of other diseases or hinder their treatment. In Italy, containment measures caused a large decline in cancer surgeries [23, 29]. Furthermore, in Spain, questions about the impact on the diagnosis of melanoma have been raised [30]. Restrictive measures have also impacted research. The Medicines and Healthcare Products Regulatory Agency in the United Kingdom has issued guidance on managing clinical trials during the pandemic [7] and has suggested the delivery of medications to patients’ homes to avoid unnecessary trips to the clinic.

Social distancing and quarantines have put millions of people at a greater risk of disrupting lifestyles that probably benefited their mental health, such as physical activity [31]. A survey in China’s Hubei province demonstrated that during confinement, there was an increase in the rates of anxiety, depression, and alcohol use [1].

Thus, to date, there remains no global consensus on applying social isolation measures regarding the trajectory of the disease. This is because the eventual number of predicted cases and deaths due to COVID-19 has been based on a few epidemiological models, which are grounded in untested assumptions about the impact of social distancing policies [10].

Accordingly, using existing mobility data, we verified that social distancing decreases the spread of the virus that causes COVID-19, but to have a positive effect, it must have a duration of up to 10 days. However, other studies discussed above highlight that social distancing actions, mainly lockdowns, must be applied in moderation because they may compromise individuals’ physical, mental, and emotional well-being.

## Conclusion

This study aimed to analyze the impact of the social isolation index on the number of transmissions and deaths due to COVID-19 in the state of São Paulo, Brazil. We selected this region as it has better quality data available and a population similar to that of many European nations. In addition, this region was where the first case and community transmission of COVID-19 in Brazil was reported to have occurred.

The use of technological geolocation tools enabled us to gauge the daily isolation index of the population in this region. Hence, our study is one of the first to apply an analytical methodology in evaluating the impact of isolation measures on the number of cases and deaths resulting from COVID-19. Our findings corroborate those of most published studies, indicating that an increase in the isolation index has positive repercussions for the number of deaths and infected individuals. This study’s contribution to the existing research is the empirical evidence ​​it provides regarding social distancing measures in the state of São Paulo.

We found that isolation had a positive effect on COVID-19 transmission. This is an important finding as it can consolidate isolation as an important prevention tool. Although the infection transmission rate in the state was 6.9%, it is important to highlight that this is the pure effect, which can be intensified with the adoption of additional non-pharmaceutical measures.

However, social distancing may cause a number of related problems, such as delays in surgeries and diagnoses as well as psychiatric issues. Despite this, given the current public health emergency, we believe that social distancing offers more advantages than drawbacks, since it can serve as a non-pharmacological tool to reduce the disease’s proliferation rate. In the same sense, long-term isolations could prevent almost 36% of deaths, demonstrating the potential positive trade-off for such measures.

The absence of a guideline to assess the quality of the results was a limitation of our study. The lack of aggregate data has limited the use of such tools. The second limitation of our study is that it was restricted to the state of São Paulo. Despite being densely populated, São Paulo may exhibit particular urban and climate characteristics that would make it difficult to generalize our findings to other regions or countries. The third limitation of our study concerns the social isolation data, which did not account for individuals who left home without carrying their devices. However, we believe that the percentage of such individuals was very small and would not have changed the results because commerce and traffic were monitored by authorities. Hence, we recommend that other regions be included in future research. In addition, a study evaluating isolation by gender should also be conducted. Moreover, we suggest the use of additional variables, such as human traffic, awareness campaigns, the distribution of personal protective equipment, and other non-pharmaceutical measures.

## Data Availability

The datasets generated and/or analysed during the current study are available in the Social Isolation Index In Loco Website repository, shorturl.at/bdqv1.

## Abbreviations

WHO: World Health Organization
VAR: Vector autoregression
